# Autonomy support in higher education: a key strategy for the well-being of university students

**DOI:** 10.12688/f1000research.144803.1

**Published:** 2024-07-26

**Authors:** DAVID PINEDA, JOSE EDUARDO LOZANO-JIMENEZ, Juan Antonio Moreno-Murcia

**Affiliations:** 1 Universidad Miguel Hernandez de Elche, Elche, Valencian Community, 03202, Spain; 2 Corporacion Universitaria de la Costa, Barranquilla, Atlantico, 080002, Colombia; 3 Universidad Miguel Hernandez de Elche, Elche, Valencian Community, 03202, Spain

**Keywords:** motivation; cohesion; University students; teaching style; autonomy support

## Abstract

**Introduction:**

Amid changing social dynamics, the world of higher education faces, among other challenges, the growing impact on the mental health of students. In this scenario, the Self-Determination Theory (SDT) highlights the important role of autonomy support as it generates positive effects on students' motivation and well-being.

**Methods:**

The present study tests the predictive capacity of the teacher’s interpersonal style of autonomy support in a higher education institution, in relation to the satisfaction of basic psychological needs, autonomous motivation and depressive symptoms. A sample composed of 356 Spanish university students of which 237 were male (66.57%) from different grades and courses, aged between 17 and 57 years ( *M* = 20.83; *SD* = 3.44), from middle socioeconomic strata, was used, and selected through purposive sampling.

**Results:**

After the analysis of structural equations, the results showed that the teacher’s interpersonal style of autonomy support positively predicted the satisfaction of basic psychological needs and this the autonomous motivation; but negatively depressive symptoms.

**Conclusions:**

The model describes the possible importance of promoting the teacher’s interpersonal style of autonomy support in higher education as a protective factor for well-being and mental health. These findings highlight the importance of motivational strategies that higher education teachers must implement to promote student motivation and well-being.

## Introduction

Higher education institutions face complex challenges in achieving quality and recognition ( Al Abduwani, 2017; Kuoppakangas *et al*., 2019; Tikhonova & Raitskaya, 2018). In addition to the challenge of being recognized as centers for the generation of knowledge, innovation and technology, they have an emerging challenge: training professionals with positive psychological capacities ( Siu *et al*., 2014), capable of advancing and concluding their training in a timely manner ( Gutiérrez *et al*. , 2019) and with this, to respond to the productive sector, which requires competent professionals with high adaptive and coping capacities in changing and challenging environments ( Moreno & Morales, 2017). These expectations find on university campuses a complex and increasingly frequent reality regarding mental health problems ( Levine *et al*., 2022).

University students are considered the future of society and their well-being and mental health are a central aspect of their training ( Wang *et al*., 2021), especially since they are a high-risk population ( Chen & Lucock, 2022). Entering the university marks the beginning of a new step in the lives of young people ( Teixeira *et al*., 2022). The transition from secondary education to university implies academic, family and personal challenges, which can become excessive pressures and worries for students, which can affect their mental health as the demand increases ( Samuel & Kamenetsky, 2022), as well as their academic success, productivity and their social relationships ( Dessauvagie *et al*., 2022).

The growing “mental health problem” in higher education impacts academic processes such as attendance, performance, engagement, and graduation ( Teixeira *et al*., 2022). Although during the Covid-19 pandemic it faced a complex scenario ( López-Núñez *et al*., 2021), today after having declared its end by the WHO, it continues to generate serious impacts.

Approximately one in five college freshmen have experienced a mental disorder that is associated with reduced academic performance, further role impairment, and increased college dropout ( Husky *et al*., 2022). Depression is one of the most common mental health conditions among this population. More than half of the students at public universities in the United States suffer from depression and anxiety ( Limone & Toto, 2022). Its symptoms can persist over time and can significantly affect the academic aspects, relationships, and daily functioning of students ( Porter, 2018).

Given this worrying scenario of mental health on university campuses, it is necessary to approach its understanding and approach ( Saha *et al*., 2022). To this end, the Self-Determination Theory (SDT), as a model that aims to understand human behavior through the influences of the context and interpersonal perceptions ( Deci & Ryan, 2008), is a key resource for the analysis of motivational processes in higher education, as a central component of student well-being ( Gillet *et al*., 2019).

### Self-determination theory

The SDT postulates that people have a natural inclination to experience a sense of choice and psychological freedom regarding their thinking and actions ( Deci, 2004; Ryan & Deci, 2017) or, in other words, the tendency towards autonomous motivation. SDT starts from the fact that motivation unfolds in a continuum of processes that go from demotivation to autonomous motivation, passing through introjected motivation and integrated motivation. On this continuum, amotivation, referring to the absence of motivation, is at the opposite end of autonomous motivation, which is self-determined in nature. In the middle is extrinsic motivation, less self-determined, which oscillates between four expressions: external regulation, introjected regulation, identified regulation, and integrated regulation ( Ryan & Deci, 2020). Focusing on autonomous motivation means starting from the natural tendency and makes it possible to promote it more effectively ( Weidinger *et al*., 2016).

On the other hand, the SDT postulates the existence of three basic psychological needs (BPN): autonomy, competence and relationship with others, determinants for motivation and well-being ( Šakan *et al*., 2020). Autonomy implies volitional aspects and the disposition of behavior based on activities oriented with the integrated sense of self and in which people perceive that they have the possibility of choice and some control over the consequences; competence is defined as the perception of feeling capable when carrying out their tasks; and the relationship with others, refers to the need of people to be meaningfully involved with others and to feel part of a group ( Ryan & Deci, 2000). Satisfying these BPN is a necessary condition for the motivation “boosting” process to be successful ( Ryan & Deci, 2020).

Based on the above, the SDT proposes that the provision of social resources by personal networks, through an interpersonal style of autonomy support, is a predictor of BPN satisfaction ( Rocchi *et al*., 2017; Ryan & Deci, 2000), which in turn is a predictor of autonomous motivation. Specifically, the interpersonal style of autonomy support is positioned as a central social trigger for the development of self-determined motivation in students ( Zamzami & Corinne, 2019) and as a key element for academic success ( Leenknecht *et al*., 2017), well-being and mental health ( Dasinger & Gibson, 2022).

When the BPN are satisfied, a more autonomous motivation is promoted and, consequently, positive effects are achieved ( Orsini *et al*., 2018). Thus, although the SDT maintains that motivation is by nature a process that comes from within, it also affirms that it can be enhanced from contexts that facilitate the satisfaction of the BPN.

### Social triggers, autonomous motivation and teacher’s interpersonal style of autonomy support

Faced with the challenge of SDT to understand human behavior from the influences of the context ( Deci & Ryan, 2008), the different motivational styles of the figures that exert some influence on other people constitute key social triggers for the satisfaction of psychological needs and, consequently, for motivation and the effects derived from it ( Haerens *et al*., 2015).

In the educational context, the way in which teachers interact with their students constitutes a central component in SDT, insofar as the interpersonal style they adopt can influence student motivation, oscillating between a more supportive one or a more frustrating one for their BPN. When these are met, students report more motivation and engagement ( Orsini *et al*., 2018; Patall *et al*., 2018) and are more likely to deeply process learning material, performing better ( Jang *et al*., 2016), higher well-being ( Gillet *et al*., 2019), higher educational aspirations, and lower levels of academic dropout ( Ketonen *et al*., 2019). When teachers support students' expression and pursuit of their personal interests and goals, they become more engaged in their learning process ( Bronson, 2016; Reeve & Shin, 2020).

Through their behavior, teachers can promote positive and adaptive behaviors in their students. When adopting an autonomy-supportive motivational style, students have more opportunities to take initiative and play a leadership role ( Vermote *et al*., 2020), as they catalyze greater autonomous motivation, curiosity, and a desire to challenge ( Ryan & Deci, 2000), achieving the satisfaction of their BPN and consequently developing greater autonomous motivation ( Frielink *et al*., 2018), a key factor for academic success and well-being ( Depasque & Tricomi, 2015; Tahrekhani & Sadeghian, 2015). In this way, with the satisfaction of the BPN of the students, the teachers make possible the choice, the perception of competence and the relationship with others ( Soenens *et al*., 2018).

When, on the contrary, teachers adopt a controlling style, based on pressure on students, which does not enhance the satisfaction of their BPN, an increase in demotivation is observed ( Martinek *et al*., 2020) and can lead to a action oriented by fear of punishment or shame ( Leenknecht *et al*., 2017), which is associated with a lack of commitment ( Jang *et al*., 2016), loss of initiative and less learning ( Ryan & Deci, 2000).

### Personal triggers – mental health

Just as the teacher is a social trigger that can promote quality motivation, the individual characteristics of students can also promote or hinder their motivation. Recent works highlight the role of non-cognitive traits in the educational setting on motivational processes; such as the temperamental, attitudinal and motivational characteristics of the student ( Fomunyam & Mnisi, 2017). These personal variables of the students interact with the teacher’s interpersonal style and must be taken into account, insofar as they can improve their own motivation, achievement and well-being ( Borae & Kim, 2017; Cortez *et al*., 2019; Nonaillada, 2019; Seong-Lee & Chen-Hsieh, 2019).

Many first-year college students experience stressors that affect their adjustment and well-being ( Samuel & Kamenetsky, 2022). About a fifth suffer from some mental disorder ( Dessauvagie *et al*., 2022). The transition to university is a stage of constant psychosocial and academic changes, in which the appearance of anxious and depressive symptoms is common ( Sit *et al*., 2022), and is added to the aggravation of previous mental health problems ( Hernández-Torrano *et al*., 2020), due to the developmental, academic, occupational, and social challenges they face.

University students present high rates of mental health problems ( Limone & Toto, 2022). Depression is one of the most common in this population ( Porter, 2018; Ruiz-Hernández *et al*., 2022), and constitutes one of the personal factors that plays a relevant role in affecting their well-being, which compromises their daily functioning and that it hinders the development of their academic career ( Levine *et al*., 2022), to the point that up to 42.7% of students felt too depressed to function regularly ( Keeler *et al*., 2021).

For this reason, it is particularly important to be attentive to depressive symptoms, since it is associated with other problems such as suicide, addiction and eating disorders. In particular, 20% of college students, adolescents, and young adults in the United States had suicidal tendencies for the year 2018, constituting the group with the highest percentage in the United States ( Limone & Toto, 2022).

Depressive symptoms among college students are higher than in the adult population and higher than their non-college peers ( Chen *et al*., 2013). This problem even persists throughout the years of their academic training to the point that 60% of the students who indicated a mental health problem at the beginning of their career reported maintaining it two years later ( Zivin *et al*., 2009). The most common depressive symptoms present as heightened negative affect, anhedonia, weight loss or gain, motor retardation, concentration problems, insomnia, restlessness, agitation, and feelings of worthlessness ( Levine *et al*., 2022).

SDT offers a key to the analysis, based on the fact that BPN are associated with motivational regulation and, consequently, with mental health ( Teuber *et al*., 2021). In this direction, various studies suggest that the negative affect and frustration of BPN contribute to the development of depressive symptoms ( Ryan & Deci, 2017; Šakan *et al*., 2020; Vansteenkiste & Ryan, 2013), especially among younger students ( Shek *et al*., 2022). Furthermore, negative affect, along with other variables such as sensitivity to anxiety, emotional regulation, tolerance for uncertainty, or perfectionism, could be at the base of numerous psychopathological disorders, especially emotional disorders ( Ferrer *et al*., 2018; Pineda, 2018; Pineda *et al*., 2018).

However, just as the frustration of BPN is a consistent predictor of both negative affect and depressive symptoms over time ( Levine *et al*., 2022; Šakan *et al*., 2020), their satisfaction, based on the implementation of a teacher’s interpersonal style that supports autonomy is a predictor of greater autonomous motivation and well-being ( Gillet *et al*., 2019).

In this framework, although the damping effect on depression of this interpersonal style has already been investigated in primary and secondary school students ( Zhang *et al*., 2022), it is necessary to analyze it with university students. On the other hand, although there is a very important precedent on the (negative) correlations between autonomy with respect to depression, anxiety and somatic symptoms ( Sheikholeslamia & Arab-Moghaddama, 2010), it is necessary to update the findings in the present time and context.

The present study, aware of the importance of the role of the teacher in academic success ( Adi Badiozaman *et al*., 2019; Langdon *et al*., 2017), and in the mental health of higher education students, aims to examine the relationships between interpersonal style of teacher autonomy support, BPN satisfaction, autonomous motivation and depressive symptoms of a group of university students, through a structural regression model.

## Methods

### Ethics

The study was conducted in accordance with the Declaration of Helsinki. The study was presented and approved by the ETHICS AND INTEGRITY COMMITTEE IN RESEARCH, VICE-RECTORATE FOR RESEARCH AND TRANSFER - MIGUEL HERNÁNDEZ UNIVERSITY OF ELCHE (approval number: DPS.DPS.230623. The participants agreed to be part of the study and gave their written informed consent. Everyone completed the questionnaires, willingly and anonymously to be able to respond honestly and sincerely.

### Participants

The sample was made up of 356 Spanish university students, 237 of whom were boys (66.60%) from different grades and courses at a Spanish university, aged between 17 and 57 (M = 20.83; SD = 3.44). In general, coming from middle socioeconomic strata. They were selected through intentional sampling, taking into account the availability of teachers at the time of administration of the instruments. Second semester students were not included.

### Measures

The present study involves the use of questionnaires that have been validated by previous studies. Below in the description of each measure is the study citation and URL. It is also included in the references section.

**Autonomy support.** To measure the interpersonal style of autonomy support perceived by Higher Education students from their teacher, the Moreno-Murcia *et al*. Autonomy Support Scale (EAA) was used ( Moreno-Murcia *et al.*, 2019). It consists of 12 items (e.g. “Provides explanations that help us understand the personal utility of carrying out said activity”) and the scale begins with an introductory heading such as: “My teacher in class…”. This is rated on a Likert scale from 1 (Strongly disagree) to 5 (Strongly agree). The reliability with the present sample, measured with the alpha and omega indices have been.94 and.94 respectively.

**Basic psychological needs.** The Spanish version of the Échelle de Satisfacción des Besoins Psychologiques in the educational context ( León *et al*., 2011) by Gillet *et al*. (2008) was used. The scale was preceded by the statement “In my class…” and made up of 15 items referring to competence (e.g. “I have the feeling of doing things well”), to autonomy (e.g. “I generally feel free to express my opinions”), and to the relationship with others (e.g. “I feel good with the people with whom I relate”). The responses were established on a Likert-type scale ranging from 1 (Strongly disagree) to 5 (Strongly agree). The internal consistency in this sample, measured with the alpha and omega indices, have been .93 and .93 for competence, .92 and 92 for autonomy, and .93 and .92 for the dimension of relationship with others.

**Autonomous motivation.** To measure student motivation, the intrinsic motivation subscales (knowledge, achievement, and stimulant) and identified motivation of the version translated and validated into Spanish by Núñez, Martín-Albo *et al*. (2005) from the Échelle de Motivation en Éducation (EME) ( Vallerand *et al*., 1989) were used. The dimensions are made up of four items each (e.g. “For the pleasure I feel by expanding my knowledge on topics that interest me”). It is preceded by the phrase “Why do you study this subject?” and the responses are collected on a Likert-type scale ranging from 1 (Totally disagree) to 7 (Totally agree). The internal consistency of the scales applied in this sample, measured with the alpha and omega indices respectively, have been .92 and .92 for knowledge, .92 and .92 for achievement, .95 and .96 for the stimulating dimension and .81 and .79 for the identified motivation.

**Depression.** To measure the depressive symptoms of the students, the PHQ-4 questionnaire ( Kroenke *et al*., 2009) was applied. The PHQ-4 is a brief, four-item Emotional Disturbance Symptom Assessment Instrument designed for use in clinical and research settings where rapid and efficient symptom assessment is required. The PHQ-4 assesses the frequency and severity of symptoms of depression and anxiety in the last two weeks, using a response scale of 0 to 3 (0 = Never, 1 = Several days, 2 = More than half of days, 3 = Almost every day). The first two items of the PHQ-4 assess the presence and severity of depressive symptoms, including sadness and lack of interest or pleasure in activities. The last two items assess the presence and severity of anxiety symptoms, including excessive worry and difficulty relaxing. The PHQ-4 has proven to be a useful and reliable tool for the assessment of depressive and anxiety symptoms in various clinical and community settings, and its short length makes it ideal for use in settings with limited time and resources ( Cano-Vindel *et al*., 2018). Internal consistency with the present sample has been .76 for Cronbach's alpha and .77 for McDonald's omega.

### Procedure

The present research has the approval of the Human Research Committee of the corresponding University, with authorization code DPS.DPS.230623 (details masked for the review process). After sharing with the Academic Department management, the teachers involved were contacted to inform them of the objective of the research and request their collaboration so that students could fill out the questionnaires in their class time. To guarantee a greater number of participants, the questionnaires were mostly administered in practical classes. The application was not made in the same subject, since none is repeated throughout the different levels of the study plan. The objective of the study and how to fill in the questionnaires were explained to them in a generic way, resolving any possible doubts that might arise during the process. In particular, the instruction was given to answer the questionnaires, not having in mind a specific subject, but rather their general experience in relation to the development of those they have taken throughout their university education. Although initially the sample was made up of more students, responses with outlier values occurred in 32 participants and it was decided to eliminate them. Once this was done, the participants agreed to be part of the study and gave their written informed consent. Everyone completed the questionnaires, willingly and anonymously to be able to respond honestly and sincerely. The time required for its completion was approximately 20 minutes.

## Results

In this study, an exploratory analysis of the data was carried out through the application of descriptive and statistical measures, to evaluate the distributions of the variables and detect possible extreme and missing values. Next, a correlation analysis was performed to assess the relationship between the variables of interest. A path analysis was then performed to examine the relationships between the variables within the proposed theoretical model, estimating the standardized regression coefficients to assess the magnitude and direction of the relationships. The model fit indices CFI, GFI, RMSEA, and SRMR were used to assess the goodness of fit of the proposed model ( Byrne, 2001; Kline, 2015). All statistical analyzes were carried out using R software version 4.2.2 ( R Core Team, 2022).

### Descriptive and correlation analysis of all variables

This section presents the results of the descriptive and correlation analysis of the data, as well as the analysis for skewness and multivariate kurtosis. The descriptive and correlation analysis of the variables has been reflected in Table 1. Regarding the results of the Mardia test, a b1p value of 17.27 was found, indicating a positive multivariate asymmetry in the data. This result is supported by the asymmetry measure, which obtained a value of 1022.09 (p < .001). In addition, the measure of asymmetry of small samples obtained a value of 1032.47 (p < .001). These results indicate that the data present a strong positive skewness in multiple dimensions. On the other hand, the value of b2p was 119.61, which suggests a positive multivariate kurtosis in the data. The kurtosis measure obtained a value of 13.8 (p < .001), which indicates a greater concentration of data around the mean, compared to the normal distribution.

**Table 1.** Means, standard deviations, and correlations with confidence intervals.

Variable	*M*	*SD*	1	2	3	4	5
1. Autonomy support	42.29	9.21					
2. Satisfaction with autonomy	15.75	5.05	.44 ** [.35, .52]				
3. Satisfaction with competence	15.53	5.09	.31 ** [.21, .40]	.37 ** [.27, .45]			
4. Satisfaction with relationships with others	28.44	7.42	.45 ** [.36, .53]	.57 ** [.50, .64]	.29 ** [.19, .38]		
5. Autonomous motivation	83.57	21.75	.42 ** [.33, .50]	.67 ** [.61, .72]	.52 ** [.44, .59]	.47 ** [.39, .55]	
6. Depressive symptoms	1.83	1.57	-.08 [-.18, .03]	-.10 * [-.21, -.00]	-.10 * [-.21, -.00]	-.16 ** [-.26, -.06]	-.09 [-.19, .01]

*Note. M* and *SD* represent the mean and standard deviation, respectively. Values in brackets indicate the 95% confidence interval for each correlation. The confidence interval is a plausible range of population correlations that could have caused the sample correlation ( Cumming, 2014).

* *p* < .05.

** *p* < .01.

### Structural regression model

**Figure 1.**
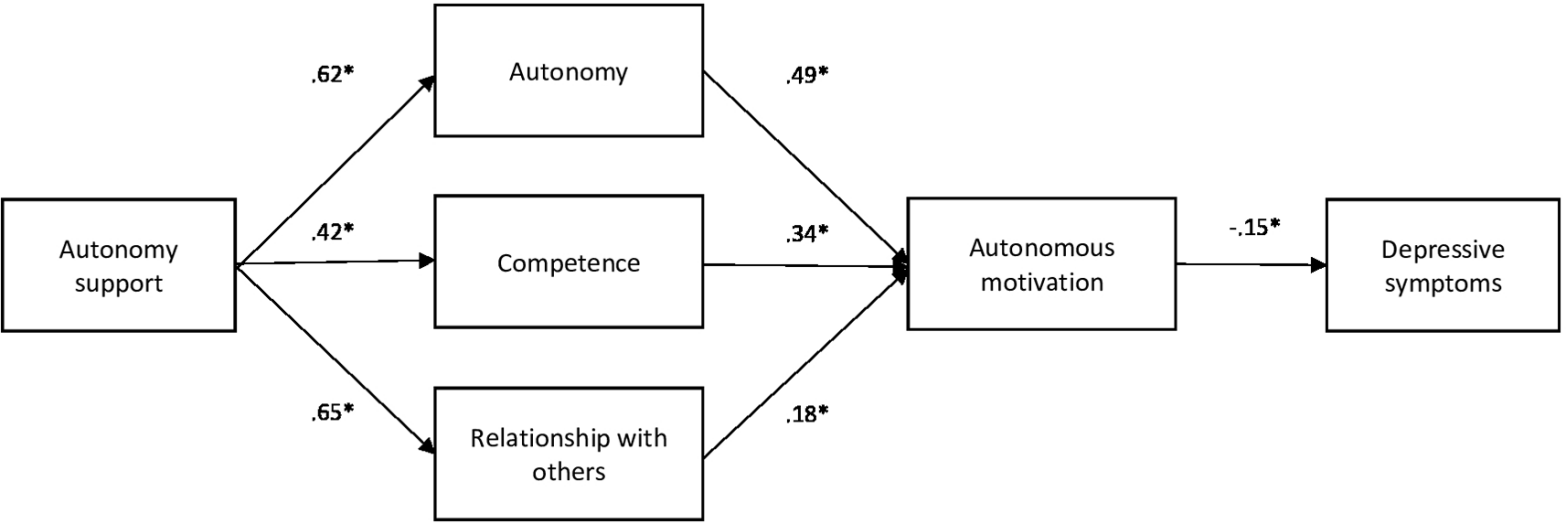
Path analysis model. *Note.* The path analysis shows the association between teacher's interpersonal style of autonomy support, satisfaction of the basic needs of the student body, autonomous motivation and depressive symptoms. The coefficients presented are the standardized regression coefficients. * *p*<.001.

An analysis was carried out with the structural equation model using the DWLS (Diagonally Weighted Least Squares) estimation method, as the most robust method to the violation of the normality assumption ( Flora & Curran, 2004). The results indicate that the model adequately fits the observed data, according to the Chi-square goodness-of-fit test statistic (χ ^2^ = 28.495, df = 8, p < .001) and other fit indices such as the global fit index (GFI = .982), the comparative fit index (CFI = .975), the root mean square error of approximation (RMSEA = .085), or the absolute mean of fit (SRMR = .080). In addition, significant relationships were found between the model variables, and the standardized regression coefficients indicate that autonomy support is positively related to autonomy satisfaction (β = .622), competence (β = .419), and interpersonal relationships (β = .648). Likewise, satisfaction with autonomy (β = .488), with competence (β = .338), and with interpersonal relationships (β = .175), predicted autonomous motivation and this, in turn, was negatively related to depression (β = -.149). These results suggest that autonomy support, autonomous motivation, as well as the satisfaction of autonomy, competence, and interpersonal relationships are important factors to consider in the prevention and treatment of depression in college students (see Figure 1).

## Discussion

The study tested a model that proposed the predictive ability of the teacher’s interpersonal style regarding depressive symptoms in college students, mediated by BPN satisfaction and autonomous motivation. All variables were positively and significantly correlated with each other, except for depressive symptoms, which were negatively correlated with BPN satisfaction and autonomous motivation. It is confirmed that the interpersonal teaching style of autonomy support positively predicts BPN satisfaction and autonomous motivation, and negatively predicts depressive symptoms in university students.

These results confirm that the teacher’s interpersonal style of autonomy support constitutes a nutrient for the satisfaction of BPN and autonomous motivation ( Deci & Ryan, 2008), as long as teachers design learning opportunities that enable decision-making, the perception of competence and the construction of positive interpersonal relationships, and, consequently, are a nutrient for academic processes ( Leenknecht *et al*., 2017) and for the well-being and mental health of university students ( Dasinger & Gibson, 2022). Various studies have reported this relationship. In particular, when the BPN are satisfied, thanks to the adoption of a motivational style of autonomy support by the teacher, students report greater autonomous motivation ( Frielink *et al*., 2018) and greater academic commitment ( Orsini *et al*., 2018; Patall *et al*., 2018) expressed in better performance ( Jang *et al*., 2016).

On the other hand, the results obtained reinforce the expectation that the motivational benefits of students are also positively related to life satisfaction, well-being and mental health, and consequently, negatively, with mental illness and depressive symptoms. Studies such as that of Gutiérrez *et al*. (2017) support this idea. In general, they conclude that classroom climates associated with interpersonal styles that support autonomy and cooperation are determinant for students to present high levels of satisfaction with life. Similar conclusions were reached by Lozano-Jiménez *et al*. (2021) in a study carried out with university students in which the predictive capacity of the teacher’s interpersonal style of autonomy support is corroborated, in relation to the satisfaction of the BPN and autonomous motivation, with satisfaction with life.

In relation to well-being and mental health Gillet *et al*. (2019) in a study carried out with French university students, concluded that well-being, in terms of positive affect, is positively related to BPN satisfaction. Teuber and Niewöhner (2021) also agree, pointing out, in their research with German higher education students, that BPN satisfaction is positively linked to well-being and negatively linked to depression. More recently Levine *et al*. (2022), concluded that BPN frustration was the only consistent predictor of both negative affect and depressive symptoms.

These results are particularly relevant, as they highlight the important role of the autonomy support style in mental health. According to Samuel and Kamenestsky (2022), students tend to use informal sources of support, such as family and friends, even with formal sources of support available through mental health services on or off campus. In this sense, teachers constitute a protective factor when in class settings they provide the necessary conditions for the empowerment of autonomous motivation as well as for the well-being of their students, especially when formal support on campus has barriers that affect access to care, either due to costs, availability and its general effectiveness ( Samuel & Kamenestsky, 2022).

It is important to consider that, although the teacher’s interpersonal style of autonomy support is a resource in higher education to contribute to the well-being of students, it should also be implemented from school education, in terms of psychopathologies identified in the early years of university began before entering higher education as documented by Auerbach *et al*. (2019).

On the other hand, this study makes contributions of a pedagogical nature in that it highlights the importance of implementing strategies to enhance student motivation and consolidate protective factors for their mental health. Allowing decision-making, promoting active participation and teamwork, and guiding students in their study processes are key strategies to consolidate greater motivation and greater well-being ( Matos *et al*., 2018; Cheon & Reeve, 2015). Within the framework of pedagogical processes, it is important to address the fact that most institutions have tight schedules and continuous study sequences, which affects student performance and their mental well-being. Hence, another challenge is that, in order to enhance the effects of the teacher’s interpersonal style of autonomy support, the campus environment must be adequate and protective ( Limone & Toto, 2022).

One of the limitations of this study is that, since it has a correlational scope, only correlations are established between the variables treated, and although the structural equation model allows a prediction to be made, it is not possible to establish a causal relationship. Experimental studies are necessary to explain the causal relationships of the variables studied, and others in which the sample is random and equally distributed by gender. In addition to the issue of scope, the type of cross-sectional design adopted does not allow an analysis to proceed over a longer timeline. This makes it necessary for subsequent studies to measure the evolution of the variables in various time cuts. Similarly, the proposed model is the one that presented the best fit, but due to the problem of equivalent models presented by the structural equations technique ( Hershberger, 2006), it is assumed that the proposed model would be only one of the possible ones.

As practical implications, in a higher education scenario, the consideration of certain personal variables of the student related to self-regulation should be elements that serve as a basis to complement and guide effective pedagogical practices based on promoting autonomy support and strengthening success-oriented academic and the promotion of students’ mental health. This represents a challenge, since according to Ryan and Deci (2020) conventional relationship styles are installed under the protection of institutional models and educational policies conventionally focused on control practices. Finally, results suggest promoting a much broader and more diverse university teacher training, focused not only on deep specialization and expertise in an academic area and on research, but also on mastering effective teaching-learning strategies ( Oriol-Granado *et al*., 2017).

As a conclusion, in the context of higher education, the teacher’s interpersonal style of autonomy support facilitates the satisfaction of basic psychological needs and, consequently, enhances autonomous motivation, which enables students to perceive depressive symptoms with less intensity.

## Data Availability

All data supporting this research are available in a public repository, in order to promote the integrity, discovery and reuse of this research. Data are available in: Dates of PIIE. 24542071 Moreno-Murcia, Juan Antonio. Dates of PIIE. figshare. Dataset. DOI 10.17605/OSF.IO/GM4JT https://osf.io/gm4jt/?view_only=ead887f5ba2345fc96ed95d1725957d7 Data is available under the terms of the Creativa Commons - CC BY 4.0 DEED Attribution 4.0 International
