# Supplementary material for: Autonomy support in higher education: a key strategy for the well-being of university students
Source: F1000Res. 2025 Aug 18;13:839. Originally published 2024 Jul 26. [Version 2] doi: 10.12688/f1000research.144803.2 (PMC12498519; doi:10.12688/f1000research.144803.2)
Supplement: Peer review history

# Reviewer report: Reviewer response for version 2

**DOI:** 10.5256/f1000research.186274.r406429

**Published:** 2025-09-23

**Authors:** Ana Velasquez

**Affiliations:** 1 Universidad de los Andes, Bogotá, Bogota, Colombia

This new version of the manuscript responds to most of the comments provided in my previuos review. However, I am still concerned about the support for the mediation interpretation. The manuscript presents a structural equation modeling (SEM) approach to test indirect effects, which is appropriate in principle; however, the description of the mediation procedure is insufficiently detailed. The authors report standardized path coefficients and p-values for indirect effects but do not specify how these p-values or confidence intervals were obtained (eg., bootstrapping,  Sobel test, or another method). This omission limits reproducibility and interpretability. I recommend adding a clear explanation of the exact method used to estimate and test indirect effects, including a citation as support for the procedure conducted.

Is the work clearly and accurately presented and does it cite the current literature?

Yes

If applicable, is the statistical analysis and its interpretation appropriate?

Partly

Are all the source data underlying the results available to ensure full reproducibility?

Partly

Is the study design appropriate and is the work technically sound?

Yes

Are the conclusions drawn adequately supported by the results?

Partly

Are sufficient details of methods and analysis provided to allow replication by others?

Yes

Reviewer Expertise:

Social and emotional development; Classroom climate

I confirm that I have read this submission and believe that I have an appropriate level of expertise to confirm that it is of an acceptable scientific standard.

# Reviewer report: Reviewer response for version 2

**DOI:** 10.5256/f1000research.186274.r406430

**Published:** 2025-09-08

**Authors:** Domenico Monacis

**Affiliations:** 1 Departmnet of Education and Sports Sciences, Pegaso University, Naples, Campania, Italy

I would congratulate authors for the enormous effort in answering all comments and suggestions. 

 The proposed revisions are adequate and useful to enhance the quality of the manuscript, the significance of the contents and a better understanding of findings. 

 I recommend the indexing of the manuscript in the present form.

Is the work clearly and accurately presented and does it cite the current literature?

Yes

If applicable, is the statistical analysis and its interpretation appropriate?

Yes

Are all the source data underlying the results available to ensure full reproducibility?

Yes

Is the study design appropriate and is the work technically sound?

Yes

Are the conclusions drawn adequately supported by the results?

Yes

Are sufficient details of methods and analysis provided to allow replication by others?

Yes

Reviewer Expertise:

physical activity; sport science; sports science;

I confirm that I have read this submission and believe that I have an appropriate level of expertise to confirm that it is of an acceptable scientific standard.

# Reviewer report: Reviewer response for version 1

**DOI:** 10.5256/f1000research.158650.r308755

**Published:** 2024-11-25

**Authors:** Ana Velasquez

**Affiliations:** 1 Universidad de los Andes, Bogotá, Bogota, Colombia

This article examined the relationship among several motivational variables and mental health (particularly depression), in a group of 356 Spanish university students. It was found that university teachers’ autonomy support predicted students’ reports of their satisfaction of basic psychological needs. The satisfaction of these needs was also a predictor of the students’ autonomous motivation, which, in turn, was negatively related with depressive symptoms. All of this was tested through structural equation models, with cross-sectional data.

 The study provides support for self-determination theory, as it shows the relevance that teachers’ autonomy support has for students’ motivation and wellbeing. The paper presents and organized rationale, which is well supported by pertinent and recent literature. The methodological design is sound enough to test for the proposed hypothesis, and the limitations stated info the discussion section acknowledge that no causal inferences may be made.

 In the introduction, second paragraph, the authors need to further explain that statement that university students are a high-risk population.

 In the discussion section, the authors state that they “tested a model that proposed the predictive ability of the teacher’s interpersonal style regarding depressive symptoms in college students, **mediated** by BPN satisfaction and autonomous motivation.” The interpretation about the mediation effect should be restated, as the analyses do not include all the steps needed to prove a mediation hypothesis. Neither the direct effects between the predictor (i.e., teachers’ autonomy support) and the dependent variables (i.e., depressive symptoms) were tested, nor the change in those effects once the meditators are included in the model were analyzed.

 As for the source data, it was provided, but a data dictionary should also be provided so that any researcher can calculate de variables an reproduce the results.

 Finally, there are some writing mistakes that should be corrected. For example:

 -Abstract/Results: "basic psychological needs and this the autonomous"

 -Introduction, Personal triggers… section, third paragraph: " which compromises their daily functioning and that it hinders”

 -Methods, Ethics, first paragraph: a closing parenthesis is missing for “(approval number…”

 Once these issues have been revised, I think the paper will be eligible of indexing in this outlet.

Is the work clearly and accurately presented and does it cite the current literature?

Yes

If applicable, is the statistical analysis and its interpretation appropriate?

Partly

Are all the source data underlying the results available to ensure full reproducibility?

Partly

Is the study design appropriate and is the work technically sound?

Yes

Are the conclusions drawn adequately supported by the results?

Partly

Are sufficient details of methods and analysis provided to allow replication by others?

Yes

Reviewer Expertise:

Social and emotional development; Classroom climate

I confirm that I have read this submission and believe that I have an appropriate level of expertise to confirm that it is of an acceptable scientific standard, however I have significant reservations, as outlined above.

## References

[1] : Autonomy support in higher education: a key strategy for the well-being of university students. *F1000Research* .2024;13: 10.12688/f1000research.144803.1 10.12688/f1000research.144803.1 PMC1249851941058928

# Response

**Published:** 2025-08-06

**Authors:** JOSE EDUARDO LOZANO-JIMENEZ

**Affiliations:** Faculty Human & Soc. Sciences, Corporacion Universitaria de la Costa, Barranquilla, ATLANTICO, Colombia

**ANSWER TO REVIEWER**

- In the introduction, second paragraph, the authors need to further explain that statement that university students are a high-risk population.

*The statement was expanded, and references were added. Page 3:*

 Increasingly young students are exposed to disruptive situations such as studying away from home, interacting with a new academic context with new pedagogical and assessment models, changing their study and rest schedules and routines, transforming their previous social networks, and exposure to psychoactive substances, among others. These changes make them a high-risk group for their mental health (Evans-Lacko et al., 2019; Girmay & Sinh, 2019; Hsiung et al., 2019; Kim et al., 2022).

  

- In the discussion section, the authors state that they “tested a model that proposed the predictive ability of the teacher’s interpersonal style regarding depressive symptoms in college students, mediated by BPN satisfaction and autonomous motivation.” The interpretation about the mediation effect should be restated, as the analyses do not include all the steps needed to prove a mediation hypothesis. Neither the direct effects between the predictor (i.e., teachers’ autonomy support) and the dependent variables (i.e., depressive symptoms) were tested, nor the change in those effects once the meditators are included in the model were analyzed.

*This observation was clarified. Page 13:*

*Additionally, indirect effects from autonomy support to depression through the mediators were significant: via autonomy satisfaction (indirect* *β* *= –.073, p = .002), via competence satisfaction (indirect* *β* *= –.050, p = .008), and via interpersonal relationships (indirect* *β* *= –.026, p = .111; non‑significant).* *The total indirect effect was β = –.083, p < .001.*

  

- As for the source data, it was provided, but a data dictionary should also be provided so that any researcher can calculate de variables an reproduce the results.

*The information is shared in an attached file.*

  

- Finally, there are some writing mistakes that should be corrected. For example:

-Abstract/Results: "basic psychological needs and this the autonomous"

 -Introduction, Personal triggers… section, third paragraph: " which compromises their daily functioning and that it hinders”

 -Methods, Ethics, first paragraph: a closing parenthesis is missing for “(approval number…”

*The document was reviewed again, and corrections were made to writing mistakes, including those pointed out by the reviewer.*

# Reviewer report: Reviewer response for version 1

**DOI:** 10.5256/f1000research.158650.r314915

**Published:** 2024-10-03

**Authors:** Domenico Monacis

**Affiliations:** 1 Departmnet of Education and Sports Sciences, Pegaso University, Naples, Campania, Italy

I first would like to congratulate the author for this interesting study, about autonomy support in higher education. It’s a considerable effort from the researchers and the constructs involved.

 It’s well written and follow the IMRAD design and describes adequately the content of the research protocol.

 It’s well written and follow the IMRAD design and describes adequately the content of the research protocol. The background of the study is clear and points out the need to further research in this field. The methods and findings are clear, and well justified as well as data analysis.

 I would suggest specifying how participants have been recruited in a more detailed way.

 Moreover, the results and discussion are well written, and present and discuss the main results of the research in adequate manner, clearly presenting the main outcomes of the research, and discussing them accordingly.

 I would like to congratulate the author for the enormous effort in doing this research and acknowledge the contribution for the understanding of the topic. We would suggest clarification of the contents above, for easier understanding, but besides that, in my opinion, should be accepted.

 Best compliments

Is the work clearly and accurately presented and does it cite the current literature?

Yes

If applicable, is the statistical analysis and its interpretation appropriate?

Yes

Are all the source data underlying the results available to ensure full reproducibility?

Yes

Is the study design appropriate and is the work technically sound?

Yes

Are the conclusions drawn adequately supported by the results?

Yes

Are sufficient details of methods and analysis provided to allow replication by others?

Yes

Reviewer Expertise:

physical activity; sport science; sports science;

I confirm that I have read this submission and believe that I have an appropriate level of expertise to confirm that it is of an acceptable scientific standard.
